# A massive dataset of the NeuroCognitive Performance Test, a web-based cognitive assessment

**DOI:** 10.1038/s41597-022-01872-8

**Published:** 2022-12-08

**Authors:** Paul I. Jaffe, Aaron Kaluszka, Nicole F. Ng, Robert J. Schafer

**Affiliations:** 1 Lumos Labs, Inc., San Francisco, CA 94108 USA; 2 Department of Psychology, Stanford University, Stanford, CA 94305 USA

**Keywords:** Human behaviour, Cognitive ageing, Databases

## Abstract

We present a dataset of approximately 5.5 million subtest scores from over 750,000 adults who completed the NeuroCognitive Performance Test (NCPT; Lumos Labs, Inc.), a validated, self-administered cognitive test accessed via web browser. The dataset includes assessment scores from eight test batteries consisting of 5–11 subtests that collectively span several cognitive domains including working memory, visual attention, and abstract reasoning. In addition to the raw scores and normative data from each subtest, the dataset includes basic demographic information from each participant (age, gender, and educational background). The scale and diversity of the dataset provides an unprecedented opportunity for researchers to investigate population-level variability in cognitive abilities and their relation to demographic factors. To facilitate reuse of this dataset by other researchers, we provide a Python module that supports several common preprocessing steps.

## Background & Summary

Cognitive assessments are an essential tool of clinical and basic researchers in psychology: they are used to make diagnoses, monitor patient outcomes, and probe fundamental questions about the human mind. While the structure and intended use of cognitive assessments varies considerably, most assessments consist of several distinct cognitive tasks or *subtests* that assay a range of cognitive abilities^1–5^. Increasingly, researchers are turning to computerized versions of these assessments due to their increased ease of administration and scoring, reduced cost, and greater design control relative to traditional pencil-paper assessments^1,6,7^. The administration of these assessments via online platforms has also enabled the acquisition of large-scale cognitive performance datasets^1,8–10^, which could in principle serve as valuable research tools for both exploratory and targeted studies. For example, the large-scale nature of these datasets may afford a sufficient sample size to conduct targeted investigations of understudied demographic subgroups or to conduct exploratory factor analysis^11^, which generally requires large sample sizes to ensure that the resulting factor solutions are stable^12^. Here we characterize and release a large dataset of the web-based NeuroCognitive Performance Test (NCPT, Lumos Labs, Inc.)—consisting of N = 757,427 unique individuals and N = 5,451,546 subtest scores—which is to our knowledge the largest publicly available dataset of its kind.

The NCPT is a brief web-based assessment that assays cognitive function in several general domains including working memory, abstract reasoning, selective/divided attention, response inhibition, arithmetic reasoning, and grammatical reasoning. The assessment consists of several distinct subtests, many of which are close analogs of established cognitive assessments such as the go/no-go task^13^, the Trail Making Test^14^, the Corsi block-tapping test^15^, Raven’s progressive matrices^16^, and others. While the NCPT platform supports the creation of custom test batteries using a subset of the available subtests, the present resource reports data from eight standardized test batteries that have each been taken by over 1000 participants (range: 1504–318,300 participants per battery).

The NCPT has been shown to have high test-retest reliability, good concordance with other cognitive assessments, and sensitivity to clinical conditions and demographic factors^1,17^. Morrison *et al*. found that the test-retest reliability of a composite score from one NCPT test battery was *r = *0.83 as assessed by the Pearson’s correlation coefficient in ~35,000 participants that took the test ~80 days apart. Other psychological assessments have been shown to have comparable test-retest reliability^2^. Additionally, both the individual subtests of the NCPT and a composite NCPT score have been shown to have good concordance with other similar cognitive assessments, in both healthy^1^ and clinical^17^ populations. Finally, consistent with many prior studies^8,9,18–21^, Morrison *et al*. demonstrated that the NCPT is sensitive to age and educational attainment.

In addition to this validation, the NCPT has been used to assess cognitive performance in a variety of contexts by several different research teams. For example, the NCPT has been used to assess the efficacy of computerized cognitive training in healthy and clinical populations^22–24^, to assess the effect of traumatic brain injury and repetitive head injury on cognitive performance^25^, and to assess changes in cognition following surgical interventions^26^. The validation of the NCPT and its usage as a general-purpose cognitive assessment reinforces the view that a large NCPT dataset would provide a useful resource to the research community.

The present resource comprehensively describes and validates the associated NCPT dataset. We detail the preprocessing steps, provide descriptions of each of the 17 cognitive tasks (i.e. subtests), and assess subtest-subtest score correlations calculated across participants as a means of technical validation. The dataset could be used to address a variety of scientific questions, both exploratory and targeted in nature. For example, researchers could examine to what extent variation in cognitive abilities can be explained by loadings onto a small set of underlying factors (e.g. using factor analysis^11^), or characterize how different cognitive domains are influenced by demographic factors. To facilitate reuse of this dataset, we provide a Python module that enables several common preprocessing steps such as removing participants with incomplete data and outlier removal. Usage notes for this module are detailed below. We note that a substantially smaller NCPT dataset (N = 4,715 individuals) was previously released as part of a cognitive training intervention study^22^.

## Methods

### Participants

The dataset contains NCPT test scores from 757,427 participants (ages 18–99, inclusive) who registered for the Lumosity cognitive training program (Lumos Labs, Inc.) and completed the NCPT as part of their user experience between 1/7/2013 and 6/29/2020 (inclusive). These participants self-reported that their preferred language was English and that their home country was the United States, Canada, Australia, or New Zealand. Participants who did not report their age, country of residence, or preferred language were not included in the dataset. Additionally, Lumosity users who were employed by Lumos Labs were excluded from the dataset. The demographic composition of these participants is shown in Table 1, separated according to NCPT test battery. The demographic composition of the general US population is shown in the rightmost column of Table 1. These data were aggregated from the 2019 United States Census Bureau’s American Community Survey (ACS) 1-year Public Use Microdata Sample using the microdata access tool available at https://data.census.gov/.

**Table 1:** Demographic composition of the NCPT data split by test battery.

	Battery 14 N = 9529	Battery 17 N = 75597	Battery 25 N = 5275	Battery 26 N = 1504	Battery 32 N = 104395	Battery 39 N = 231954	Battery 50 N = 318300	Battery 60 N = 10873	2019 ACS N = 2599171
**N, Male**	4850 (50.9%)	30536 (40.39%)	2253 (42.71%)	689 (45.81%)	43709 (41.87%)	89553 (38.61%)	122805 (38.58%)	3819 (35.12%)	1259569 (48.46%)
**N, Female**	4572 (47.98%)	40197 (53.17%)	2885 (54.69%)	733 (48.74%)	50955 (48.81%)	116400 (50.18%)	177425 (55.74%)	6452 (59.34%)	1339602 (51.54%)
**N, Not reported**	107 (1.12%)	4864 (6.43%)	137 (2.6%)	82 (5.45%)	9731 (9.32%)	26001 (11.21%)	18070 (5.68%)	602 (5.54%)	N/A
**Ages 18–39**	7476 (78.46%)	25323 (33.5%)	3119 (59.13%)	687 (45.68%)	40365 (38.67%)	82299 (35.48%)	111656 (35.08%)	3008 (27.66%)	852603 (32.8%)
**Ages 40–59**	1776 (18.64%)	28882 (38.21%)	1595 (30.24%)	553 (36.77%)	38392 (36.78%)	89768 (38.7%)	118628 (37.27%)	4079 (37.51%)	825574 (31.76%)
**Ages 60–99**	277 (2.91%)	21392 (28.3%)	561 (10.64%)	264 (17.55%)	25638 (24.56%)	59887 (25.82%)	88016 (27.65%)	3786 (34.82%)	920994 (35.43%)
**Age (mean ± s.d. years)**	30.66 ± 12.11	47.89 ± 16.35	38.03 ± 14.86	42.87 ± 15.46	45.91 ± 16.45	46.9 ± 16.2	47.41 ± 16.62	50.86 ± 16.65	N/A
**Some high school**	457 (4.8%)	1151 (1.52%)	103 (1.95%)	12 (0.8%)	1562 (1.5%)	3262 (1.41%)	5535 (1.74%)	263 (2.42%)	171472 (6.6%)
**High school**	1779 (18.67%)	6327 (8.37%)	559 (10.6%)	128 (8.51%)	8630 (8.27%)	18234 (7.86%)	28659 (9.0%)	1202 (11.05%)	709575 (27.3%)
**Some college**	2631 (27.61%)	15130 (20.01%)	1384 (26.24%)	300 (19.95%)	21119 (20.23%)	43836 (18.9%)	64730 (20.34%)	2156 (19.83%)	569488 (21.91%)
**Associate’s degree**	601 (6.31%)	4299 (5.69%)	216 (4.09%)	97 (6.45%)	4779 (4.58%)	10191 (4.39%)	13771 (4.33%)	455 (4.18%)	217680 (8.37%)
**College degree**	2067 (21.69%)	22637 (29.94%)	1615 (30.62%)	511 (33.98%)	30786 (29.49%)	67980 (29.31%)	96748 (30.4%)	3090 (28.42%)	507242 (19.52%)
**Professional degree**	608 (6.38%)	5165 (6.83%)	294 (5.57%)	79 (5.25%)	6945 (6.65%)	14851 (6.4%)	21722 (6.82%)	783 (7.2%)	55376 (2.13%)
**Master’s degree**	792 (8.31%)	12311 (16.29%)	706 (13.38%)	231 (15.36%)	16049 (15.37%)	35040 (15.11%)	51602 (16.21%)	1640 (15.08%)	227364 (8.75%)
**Ph.D.**	112 (1.18%)	2009 (2.66%)	122 (2.31%)	28 (1.86%)	2558 (2.45%)	5576 (2.4%)	8308 (2.61%)	266 (2.45%)	37998 (1.46%)
**Other**	334 (3.51%)	1262 (1.67%)	94 (1.78%)	27 (1.8%)	1615 (1.55%)	3852 (1.66%)	6043 (1.9%)	270 (2.48%)	102976 (3.96%)
**Not reported**	148 (1.55%)	5306 (7.02%)	182 (3.45%)	91 (6.05%)	10352 (9.92%)	29132 (12.56%)	21182 (6.65%)	748 (6.88%)	N/A

The rightmost column shows demographic information for the general US population (from the 2019 United States Census Bureau’s American Community Survey (ACS) 1-year Public Use Microdata Sample). Rows with N/A indicate that the data were not available.

The data included here were collected during the participants’ normal use of a feature of the Lumosity program. In the Lumosity Privacy Policy (www.lumosity.com/legal/privacy_policy), all participants agreed to the use and disclosure of non-personal data (e.g. de-identified or aggregate data) for any purpose, meaning consent for specific uses of data, such as within this dataset, were not required. The analysis and release of the de-identified data were determined by WCG IRB (www.wcgirb.com) to be exempt under 45 CFR § 46.104(d)(4). In sum, we complied with all relevant ethical regulations.

The fact that participants in this study are all registered Lumosity users, and the fact that they self-selected to complete the NCPT, could in principle have compromised the representativeness of our sample. Indeed, as can be seen in Table 1, the demographic composition of the US general population differs somewhat from that of the NCPT sample. For example, the NCPT sample has a somewhat higher ratio of women to men and has a higher fraction of participants with college and advanced degrees than does the US general population. However, since complete demographic information (age, education, gender) is provided for the vast majority of participants in the dataset, researchers can readily control for demographic effects in their analyses. Nonetheless, it remains possible that the participants in this study differ from the general population along dimensions that are not captured in the dataset. For example, the prevalence of particular psychiatric conditions among Lumosity users could be different from that of the general population. Since we do not have information on medical or psychiatric conditions for the vast majority of participants in the dataset, we did not exclude participants based on particular diagnoses. Finally, while the fact that participants self-selected to be in this dataset may have compromised its representativeness, it is difficult to imagine a cognitive (or other) assessment study in humans that would not involve some degree of self-selection, and we have no reason to believe that the magnitude of self-selection biases in our dataset are more significant.

### Lumosity cognitive training program

Participants in this sample were registered users of Lumosity. Here we describe some of the general features of Lumosity and their relation to the NCPT. Lumosity is an online cognitive training program that includes ~50–70 games challenging a range of cognitive abilities including working memory, selective attention, problem solving, and mental flexibility. The exact number of games available to participants varied since games were added and removed from the program over time. Many of the games are inspired by established tasks used in psychology research, though the degree of correspondence varies from game to game. Detailed descriptions of the games can be found in prior publications^22,27^.

While the Lumosity games and the NCPT subtests overlap to some extent in terms of the general cognitive domains that are challenged, in our view none of the NCPT subtests constitute the same task (a specific set of stimuli, responses, and rules) as any of the Lumosity games. For example, one general difference between the Lumosity games and NCPT subtests is that the stimuli themselves and background animations are visually distinct (i.e., are not shared by any game/subtest pairs).

Finally, researchers should be aware that cognitive training with Lumosity has been shown to improve performance on some of the NCPT subtests^22,23^. However, the magnitude of this effect increases with the amount of Lumosity training^22^, and the median number of pre-NCPT Lumosity gameplays in the present dataset (median = 25) is small relative to that of prior studies that demonstrated effects of cognitive training (median number of hours engaging with Lumosity = 12.2 or roughly 500 gameplays^22^). Thus, in our view, effects on NCPT performance due to Lumosity training are unlikely to impact general conclusions that could be drawn using this dataset (e.g., the effects of demographics on cognitive performance or the correlation/factor structure of different cognitive domains).

### NCPT Overview

The NCPT consists of several brief subtests that are based on established cognitive tasks used in neuropsychology research. The assessment is self-administered, takes 20–30 minutes to complete, and is accessed through a web browser. The dataset accompanying this manuscript contains test records from eight different test batteries, each of which is composed of a different complement of subtests (5–11 subtests). For a given test battery, the order and identity of the subtests was fixed, and was therefore not adjusted based on any given participant’s behavior (e.g., their interaction with Lumosity). Aggregating across all test batteries, there are 17 distinct cognitive tasks (subtests), some of which have two different associated versions (described below). Each subtest is preceded by a brief tutorial that is designed to ensure a minimum level of proficiency with the task. Detailed descriptions of each subtest are provided below. A summary of the subtest information—including the subtest ID, cognitive task, and different associated versions—is shown in Table 2. Additional details may be found in prior publications^1,22^.

**Table 2:** Description of each of the NCPT subtests.

	Task	Short name	Version	Other versions
**Subtest ID 26**	Go/no-go	Go/no-go	v1	32
**Subtest ID 27**	Divided visual attention	DVA	v1	None
**Subtest ID 28**	Forward memory span	Forward mem.	v1	43
**Subtest ID 29**	Arithmetic reasoning	Arithmetic	v1	None
**Subtest ID 30**	Grammatical reasoning	Grammar	v1	None
**Subtest ID 31**	Progressive matrices	Matrices	v1	None
**Subtest ID 32**	Go/no-go	Go/no-go	v2	26
**Subtest ID 33**	Reverse memory span	Reverse mem.	v1	44
**Subtest ID 36**	Verbal list learning	Lists	v1	None
**Subtest ID 37**	Delayed verbal list learning	Delayed lists	v1	None
**Subtest ID 38**	Digit symbol coding	Digit symbol	v1	45
**Subtest ID 39**	Trail making part A	Trails A	v1	None
**Subtest ID 40**	Trail making part B	Trails B	v1	None
**Subtest ID 43**	Forward memory span	Forward mem.	v2	28
**Subtest ID 44**	Reverse memory span	Reverse mem.	v2	33
**Subtest ID 45**	Digit symbol coding	Digit symbol	v2	38
**Subtest ID 51**	Scale balance	Scale balance	v1	None
**Subtest ID 52**	Posner cueing	Posner	v1	None
**Subtest ID 53**	Complex memory span	Complex mem.	v1	None
**Subtest ID 54**	Object recognition	Object recog.	v1	None
**Subtest ID 55**	Dual search	Dual search	v1	None

#### Test procedure

Participants were invited via e-mail and/or an in-app prompt to take the NCPT as part of their Lumosity user experience. Participation was voluntary, was not required for continued use of Lumosity, and was not rewarded with any type of compensation. Participants completed the NCPT over a web browser from a PC and were free to choose when and where to take the test (tablet NCPT data are not included in this dataset).

#### Test batteries

A test *battery* is defined by a particular set of subtests that are administered together. This dataset includes NCPT scores from eight distinct test batteries consisting of 5–11 subtests. The subtests that were included in each battery and the order that they were administered is shown in Table 3.

**Table 3:** The subtests that make up each test battery.

	Battery 14	Battery 17	Battery 25	Battery 26	Battery 32	Battery 39	Battery 50	Battery 60
**Subtest IDs**	29, 28, 30, 27, 26	29, 28, 30, 27, 32, 31	29, 28, 33, 30, 27, 32, 31	36, 39, 40, 29, 28, 33, 30, 27, 32, 38, 37	29, 38, 28, 33, 39, 40, 30, 27, 31	29, 38, 28, 33, 39, 40, 30, 31	29, 45, 43, 44, 39, 40, 30, 31	54, 52, 53, 55, 51

Subtests are listed in the order that they were administered.

### NCPT subtests

#### Go/no-go

The go/no-go task is a well-established paradigm for assessing response inhibition^13^. In the NCPT go/no-go subtest, participants are shown a sequence of stimuli (cartoon pieces of fruit), one of which is designated as the target stimulus. Participants are instructed to respond with a key press when shown the target stimulus (go trials) and to withhold responses for all other stimuli (no-go trials). The subtest continues until participants respond correctly on 10 go trials or respond incorrectly on three trials of either type. If the participant responds incorrectly on three trials, the participant is required to complete the practice session again at least once before attempting the actual subtest again. Each stimulus is shown for a maximum of 1500 ms. If the participant fails to respond within 1500 ms on go trials, the response is considered incorrect. The subtest score is the mean response time (RT) on correct go trials in milliseconds.

#### Notes on subtest versions

Two different versions of the go/no-go subtest are included in the dataset: subtest ID 32, as described above, and subtest ID 26, which differs from the above description only in that the subtest concludes when the participant responds correctly to five trials instead of 10 trials.

#### Divided visual attention

This subtest combines aspects of useful field of view tasks^28^ and divided visual attention tasks^29^. Participants are instructed to attend to two target letters and ignore two distractor letters that are spread across the field of view. On each trial, a fixation cross is briefly displayed at the center of the browser window, followed by the four letters. Each letter is displayed within a small circle; the four letters are randomly dispersed across the visual field. After a brief presentation, the letters are obscured to prevent visual afterimage effects. After the letters and masking stimuli disappear, participants must click on the two circles that contained the target letters. To titrate the difficulty of the task, the presentation time is decreased or increased by a small amount depending on whether the participant answered the previous trial correctly or incorrectly, respectively (presentation time for the first trial: 1 s). The subtest score is the number of correct trials out of 12 total trials.

#### Notes on subtest versions

Subtest ID 27 is the only version of this subtest included in the dataset.

#### Forward memory span

This subtest assesses visuo-spatial working memory and is based on the Corsi block-tapping test^15^. On each trial, participants view a set of 10 blue circles placed at random, non-overlapping spatial locations. Following a brief delay, a random subset of circles is highlighted sequentially in orange. Each circle is highlighted for 333 ms, with a 333 ms delay between successive circles. Participants are instructed to repeat the sequence by clicking on the same circles in the order that they were highlighted. The length of the highlighted sequence (span) increases by one after every three trials, starting with a span of three on the first trial. The subtest concludes when the participant responds incorrectly on two trials at the same span level. The subtest score depends on the subtest version. For subtest ID 43, the score is the total number of correct trials. For subtest ID 28, the score is the maximum span level for which the participant answered at least one trial correctly.

#### Notes on subtest versions

Two different versions of the forward memory span subtest are included in the dataset: subtest ID 43, as described above, and subtest ID 28. In addition to being scored differently, subtest ID 28 differs from the above description in two ways. First, each circle is highlighted for 500 ms, with a 500 ms delay between successive circles. Second, the span increases by one after every two trials (rather than every three trials).

#### Reverse memory span

This subtest also assesses visuo-spatial working memory and is based on a reversed version of the Corsi block-tapping test^15^. The stimuli and general setup of this subtest are the same as for the forward memory span subtest. However, rather than report the highlighted sequence in the same order it was shown, participants are instructed to report the sequence in reverse order. All other aspects of the subtest are the same as the forward memory span subtest. The subtest score depends on the subtest version. For subtest ID 44, the score is the total number of correct trials. For subtest ID 33, the score is the maximum span level for which the participant answered at least one trial correctly.

#### Notes on subtest versions

Two different versions of the reverse memory span subtest are included in the dataset: subtest ID 44, which has the same parameters as the forward memory span subtest ID 43, and subtest ID 33, which has the same parameters as the forward memory span subtest ID 28.

#### Arithmetic reasoning

This subtest assesses arithmetic problem-solving ability and is similar to arithmetic tasks used in other studies in which questions are posed verbally rather than with Arabic numerals^30^. Participants are shown simple arithmetic problems written out in words (e.g. “two plus eight = ”) and are instructed to respond quickly and accurately by entering the correct numeric solution on the keypad. The subtest score is the total number of correct responses completed in 45 seconds.

#### Notes on subtest versions

Subtest ID 29 is the only version of this subtest included in the dataset.

#### Grammatical reasoning

This subtest evaluates logical reasoning ability and is inspired by Baddeley’s grammatical reasoning test^31^. On each trial, participants are shown a blue square and a blue triangle side-by-side, with a logical statement written underneath (e.g., “The triangle is to the left of the square”). Participants must indicate if the statement is true or false. The probability that the statement includes a negation is 50%. The subtest score is the number of correct responses minus the number of incorrect responses completed in 45 seconds, with a floor of zero.

#### Notes on subtest versions

Subtest ID 30 is the only version of this subtest included in the dataset.

#### Progressive matrices

This subtest is based on Raven’s Progressive Matrices and measures abstract reasoning ability^16^. In the NCPT version of this subtest, the participant is shown a 3 × 3 grid with abstract patterns in the eight upper-left squares. The task on each trial is to select which of six possible choices best completes the pattern in the grid, corresponding to the lower right square of the 3 × 3 grid. The subtest consists of 17 trials that increase in difficulty. If the participant answers three consecutive trials incorrectly, the subtest ends. The subtest score is the total number of correct trials.

#### Notes on subtest versions

Subtest ID 31 is the only version of this subtest included in the dataset.

#### Verbal list learning

This subtest is based on the Hopkins Verbal Learning Test-Revised (HVTL-R) and assesses episodic verbal learning and memory^32^. Participants are presented with a list of 12 words, one at a time (each word presented for 1 s). The list is presented three times in total. After the last word is presented in each round, participants are asked to type as many of the words as they can remember. The subtest score is the total number of words correctly recalled in the final (third) round.

#### Notes on subtest versions

Subtest ID 36 is the only version of this subtest included in the dataset.

#### Delayed verbal list learning

This subtest assesses delayed recall using the same list of words as the verbal list learning subtest. After a delay of ~20 minutes, participants are asked to recall as many of the words from the presented list as they can remember. In the interim, participants complete the other subtests from the test battery. The subtest score is the total number of correctly recalled words.

#### Notes on subtest versions

Subtest ID 37 is the only version of this subtest included in the dataset.

#### Digit symbol coding

This subtest is derived from the Digit Symbol Substitution Test and measures information processing speed, particularly as it relates to visual search and memory^33^. Participants are shown a legend with the numbers one to nine mapped to abstract symbols. The legend is displayed throughout the task. On each trial, participants are shown one of the symbols and must enter the corresponding number as quickly as possible. The subtest score is the number of correct trials completed in 90 seconds.

#### Notes on subtest versions

Two different versions of the digit symbol subtest are included in the dataset: subtest ID 38 and subtest ID 45. There are no substantive differences between these two versions.

#### Trail making A

The NCPT trail making subtests are computerized versions of the Trail Making Test that is commonly used in neuropsychology research^14^. This subtest measures information processing speed. In part A of this subtest, blue circles numbered from 1 to 24 are arranged in one of six possible layouts. The task is to click the circles sequentially as quickly as possible. If the participant clicks on an incorrect number, an ‘X’ mark appears, and they must go back to the previous circle. The subtest score is the amount of time taken to click all the numbers in seconds.

#### Notes on subtest versions

Subtest ID 39 is the only version of this subtest included in the dataset.

#### Trail making B

In part B of the NCPT trail making subtest, blue circles numbered from 1 to 12 are interspersed with another set of 12 circles labeled with the capital letters A to L. The participant must click the circles with the numbers and letters in order, alternating between numbers and letters after each circle is selected. The subtest score is the amount of time taken to click all the circles in seconds.

#### Notes on subtest versions

Subtest ID 40 is the only version of this subtest included in the dataset.

#### Scale balance

The scale balance subtest assesses logical and mathematical reasoning ability and is based on the Figure Weights task introduced in the WAIS-IV^34^. On each trial, participants are shown three scales balanced by sets of simple shapes. Both sides of the two leftmost scales are filled with shapes, while the right side of the rightmost scale shows only a question mark, indicating a query that the participant must respond to. The participant must select the set of shapes that will balance the scale from five possible options. There are 25 total trials that become progressively more difficult; participants have ten minutes to answer as many trials as they can. Three incorrect responses in a row automatically ends the subtest. The subtest score is the number of correct responses.

#### Notes on subtest versions

Subtest ID 51 is the only version of this subtest included in the dataset.

#### Posner cueing

This subtest is based on the Posner cueing task which measures visual attention^35^. In the NCPT version of the task, participants are instructed to fixate on a small plus sign at the center of the browser window flanked on both sides by empty boxes. On each trial, a cue (directional arrow) is shown in place of the fixation symbol indicating to which side participants should focus their attention. Following a brief random delay, a stimulus (star symbol) is presented in one of the boxes. The participant must indicate the location of the stimulus (left or right) with a key press as quickly as possible. The stimulus remains on the screen for six seconds or until the participant responds, whichever occurs first. On 60% of trials, the stimulus is shown in the cued location; on the remaining trials, the stimulus is shown in the uncued location. The subtest score is the number of correct trials out of 100 total trials.

#### Notes on subtest versions

Subtest ID 52 is the only version of this subtest included in the dataset.

#### Complex span

The complex span subtest is a working memory task in which participants must monitor and remember two types of information concurrently. This subtest is inspired by other complex span tasks and memory updating tasks commonly used to assess working memory capacity^36,37^. In the NCPT version of the task, participants are shown a random sequence of single letters (excluding vowels) and digits (excluding zero) in which the letters and digits alternate. Following the sequence, participants are asked to report both the sum of the digits and the sequence of letters in reverse order. The first trial is a sequence of four characters (span = 4). The span increases by one if the participant responds correctly, and the subtest ends when the participant responds incorrectly twice at the same span level. The subtest score is the total number of correct trials.

#### Notes on subtest versions

Subtest ID 53 is the only version of this subtest included in the dataset.

#### Object recognition

This subtest assesses visual pattern recognition memory and is loosely based on the pattern recognition memory task of the Cambridge Neuropsychological Test Automated Battery^38^ (CANTAB). In the NCPT version of this task, participants are shown a sequence of 20 simple shapes that they are instructed to remember. Each shape is shown for 2 seconds. At the end of the sequence, the participant is prompted with 40 yes/no questions asking if a particular shape appeared in the sequence. The shapes for 20 of the questions correspond to shapes in the sequence; the other 20 do not. The subtest score is the number of correct responses.

#### Notes on subtest versions

Subtest ID 54 is the only version of this subtest included in the dataset.

#### Dual search

This subtest is a visual search task inspired by useful field of view tasks^28^ and divided visual attention tasks^29^. Participants are instructed to fixate on a cross at the center of the browser window. After a brief delay (500 ms), five randomly rotated black letters are displayed at random locations within a radius of 1/6 of the window height. The letters are sampled randomly from the following sets: LLLLL, TTTTT, LLLLT, TTTTL. After 100 ms, a red letter (L or T) appears for 30 ms in a random location along a circle with radius of 85% of the window height. The letters in the center remain for an additional 100 ms. Following the stimuli, participants are prompted with one of two randomly selected questions: 1) Were all five black letters in the center the same? [Yes/No]; 2) Which red letter appeared? [L/T]. If the participant does not respond within 6 seconds, the subtest continues, and that trial is marked incorrect. Participants complete 50 trials in total. The subtest score is the number of correct trials.

#### Notes on subtest versions

Subtest ID 55 is the only version of this subtest included in the dataset.

### Preprocessing

#### Data inclusion criteria

Only the first NCPT assessment from each participant is included in the dataset. Participants who paused for more than 24 h between successive subtests of a given test battery were excluded from the dataset (N = 72,521 participants). We excluded a small number of participants who started one NCPT test, paused and started a different NCPT test, then returned to and completed the initial NCPT test (N = 39 participants). We also excluded all NCPT data from a small number of participants who took longer than 15 minutes to complete the trail making subtests (subtest IDs 39 and 40; N = 22 participants). No other outlier filtering was done. We note that the participants in this dataset may have engaged in the Lumosity cognitive training program which can influence performance on the NCPT^22,23^. We did not exclude participants based on their engagement with Lumosity.

#### Other preprocessing steps

To comply with the *Safe Harbor* method of de-identification^39^, the age of all participants over the age of 89 was coded as 90.

#### Grand index

The dataset includes a composite Grand Index (GI) score that measures the overall performance for a given NCPT battery relative to the general population. The GI is only reported for participants that completed all subtests of a given test battery.

To calculate the GI for a given NCPT test battery, we first compute the mean of the normalized subtest scores from that test battery, where the normalized scores are calculated using the census-reweighted inverse normal transformation (INT)^40,41^ described below. These mean normalized scores are then mapped to a normal distribution centered at 100 with a SD of 15, again using the census-reweighted INT described below. Thus, a GI of 100 indicates average performance with respect to the general population.

#### Score normalization

For the purpose of calculating the GI composite score, subtest scores are first normalized using a novel reweighted INT that approximates norms from a population with the same demographic composition as the general population (described below). Scores normalized in this way indicate the participant’s performance relative to the general population. Participants who did not report their age or education, who had logged more than 25 Lumosity gameplays prior to taking the NCPT, or who did not complete the entire test battery were excluded from the sample used to calculate the norms. Subtests for which performance decreases as the raw score increases were negated prior to normalization, so that performance always increases as the normalized score increases (note that the raw scores are reported as is, without negation). These assessments are the go/no-go subtests (IDs 26 and 32) and the trail making subtests (IDs 39 and 40). Normalized scores for different versions of the same subtest are calculated separately.

The census-reweighted INT approximates normalized scores from a population with the same demographic composition as the 2019 United States Census Bureau’s American Community Survey (ACS) 1-year Public Use Microdata Sample. In particular, we use the NCPT dataset *X*={*x*_1_,…,*x*_*N*_} of assessment scores and associated demographic information to estimate normalized scores from a dataset X* with the same demographic composition as the 2019 ACS dataset, allowing for the possibility that the demographics of *X* and X* differ. These scores indicate performance relative to the general US population and are not guaranteed to be the same as normalized scores calculated using the standard INT^40,41^ since the demographics of the general population and those of our dataset are different (Table 1). This normalization procedure also adjusts for demographic differences between different test batteries, facilitating a comparison of the GI across batteries.

To calculate the census-reweighted INT, the dataset *X* is binned into *K* demographic bins (described below), where we denote the proportion of participants within demographic bin *k* as *f*_*k*_. These proportions are compared to a reference population X* derived from the ACS dataset with the same *K* demographic bins and analogous proportions ${f}_{k}^{\ast }$. For a participant *i* in demographic bin *k*, we calculate a reweighted percentile *w*_*i*_ according to:

$${w}_{i}=\frac{{f}_{k}^{\ast }}{N{f}_{k}},$$

where *N* is the total number of scores in the dataset. *w*_*i*_ can be thought of as a reweighted “width” of the corresponding percentile bin in the INT, where in the standard/unmodified INT this width is simply 1⁄*N*. Rather than use the ordinal rank of score *i* as an input to the quantile function in the INT, we calculate a reweighted rank variable ${\widetilde{r}}_{i}$ according to:

$${\widetilde{r}}_{i}=\frac{{w}_{i}}{2}+\sum _{j < i}{w}_{j},$$

where the scores have been rank-ordered so that the index *i* implicitly represents the ordinal rank of score *i*. The 0.5 weight multiplier is a correction introduced to center the percentile bins as is commonly done when applying a standard INT^40^. The reweighted rank variables ${\widetilde{r}}_{i}$ are used to determine the normalized score *y*_*i*_ according to:

$${y}_{i}={\Phi }^{-1}\left({\widetilde{r}}_{i}\right),$$

where Φ^−1^ is the quantile function of a normal distribution with mean = 100 and SD = 15.

#### Demographic bins and US Census Bureau data

For the census-reweighted INT described above, participants were binned according to self-reported age (six categories) and self-reported educational attainment (three categories) for a total of *K* = 18 total demographic bins. The age bin boundaries are: [18–29], [30–39], [40–49], [50–59], [60–69], and [70–99], where each bin includes its upper and lower boundaries. The educational attainment categories are: [some high school, high school diploma/GED], [some college, college degree, associate’s degree], and [professional degree, master’s degree, Ph.D.]. The demographic composition of the general US population defined by the same bins was determined using the 2019 United States ACS sample described above.

#### Normative data

In addition to the raw data, we provide normative data aggregated according to age, educational attainment, and gender as separate csv-files (see Data Records). Norms are calculated within each demographic bin using the same bin definitions as were used for score normalization (described above) with an additional partition based on gender (36 total demographic bins). Norms are only reported for test batteries that had at least 20 participants in each demographic bin for each subtest (batteries 17, 32, 39, 50, and 60). Participants that did not provide complete demographic information (age, education, gender) were excluded from the norm sample. For each demographic bin, we report the mean score, the score standard deviation (SD), the number of participants, and the 10^th^, 25^th^, 50^th^, 75^th^, and 90^th^ score percentiles. All summary statistics are calculated on the raw scores. These summary statistics are also reported for the GI from each test battery. For all subtests, including those with scores reported in units of time, a higher percentile indicates better performance.

## Data Records

The de-identified NCPT dataset is available without restriction on Zenodo^42^. The dataset is licensed under the Creative Commons Attribution 4.0 International license (CC BY 4.0). The raw NCPT scores and demographic information of each participant are organized into separate csv-files for each of the eight test batteries in the dataset (e.g. “battery14_df.csv”). Each file has N observation rows and 11 feature columns, where N is the total number of subtest scores, pooling across all participants. For a given participant’s data, the subtest scores are sorted in the order that they were administered. The name of each column variable and corresponding description is provided in Table 4. The self-reported educational attainment levels are coded numerically; the correspondence between the numeric education levels and a verbal description is provided in Table 5. The ‘gender’, ‘education_level’, and ‘grand_index’ fields may contain missing data. Missing values will be coded as NaN if the data are read using the read_csv function from the pandas Python package (recommended; this is used by the provided Python module described below). A NaN value for the ‘grand_index’ field indicates that the participant did not complete the entire test battery since valid scores for each subtest are required to calculate the GI. The other feature variables do not have missing data.

**Table 4:** Feature variable names in the NCPT csv-files.

Variable name	Description
user_id	Participant (user) identifier
age	Age of participant (years)
gender	Gender of participant
education_level	Numeric identifier for participant’s education
country	Home country of participant
test_run_id	Identifier for the entire test taken by the participant
battery_id	NCPT battery identifier
specific_subtest_id	NCPT subtest identifier
time_of_day	Time of day that test was initiated in hours (rounded down)
raw_score	Raw score for the subtest
grand_index	Composite score for the entire test (Grand Index)

**Table 5:** Coding of the self-reported educational attainment levels.

Education level	Description
1	Some high school
2	High school diploma/GED
3	Some college
4	College degree
5	Professional degree
6	Master’s degree
7	Ph.D.
8	Associate’s degree
99	Other

Normative data for test batteries 17, 32, 39, 50, and 60 are provided in separate csv-files (e.g. “battery17_norms.csv”). Each row contains the normative data for a specific subtest/GI and demographic bin. Each table has 13 columns: subtest_name, specific_subtest_id, age, education_level, gender, N (the number of participants in that bin), mean, SD, 10th_perc, 25th_perc, 50th_perc, 75th_perc, and 90th_perc.

## Technical Validation

### Subtest-subtest correlations

**Fig. 1:**
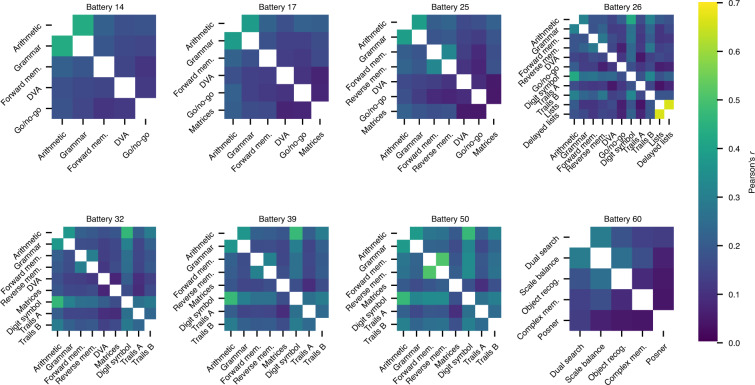
Subtest-subtest residual score correlation matrices split by test battery, as measured by Pearson’s *r*. Correlations were calculated using demographics-corrected residual scores as described in the text. Only participants who completed the entire test battery and who provided complete demographics information were included in these analyses.

One of the most robust findings in the cognitive assessment literature is that performance on different cognitive tasks tends to be positively correlated across individuals^1,43–46^. We therefore sought to verify that this ‘positive manifold’ of correlations was present in the NCPT subtests. As a preprocessing step, we first corrected for demographic effects by regressing out age, educational attainment, and gender from the raw scores. In particular, we fitted a linear regression model to the data from each subtest with the raw score as the outcome variable and age, education, and gender as predictor variables (education and gender were treated as categorical predictors, and models were fitted by ordinary least squares regression). The predictions from these models were subtracted from the raw scores to obtain demographics-corrected residual scores. Figure 1 shows the Pearson correlation coefficient (Pearson’s *r*) between the residual scores of each subtest, split out by test battery. Broadly consistent with prior reports, we observed that the score correlations were exclusively positive (min: 0.037, max: 0.66). Additionally, the magnitude of these correlations was comparable to those observed in other cognitive assessments^43–46^.

Another noteworthy characteristic of the correlation matrices shown in Fig. 1 is that the correlation structure is largely stable across different test batteries, even though each battery was completed by a different set of individuals and—in some cases—was associated with different versions of the same subtest. For example, for test batteries that included the arithmetic and grammatical reasoning subtests (all except battery 60), the correlation between these two subtests was between 0.31–0.43. Similarly, for test batteries that included both the digit symbol subtest and the trail making subtests (batteries 26, 32, 39, 50), the correlation between the digit symbol scores and either of the trail making subtests (A/B) was between 0.18–0.32. For the batteries with more than 100,000 participants (32, 39, 50), the range of the digit symbol/trail making correlations was even more narrow (0.26–0.30). This latter observation is especially noteworthy given that the version of the digit symbol subtest used in battery 50 was different from the version used in the other test batteries, suggesting that the different versions of this subtest did not substantially affect performance. In summary, the stability of the subtest correlations across the different test batteries validates the NCPT dataset as a resource that can be used to identify robust population-level characteristics of cognitive abilities.

## Usage Notes

We created a simple Python package, lumos-ncpt-tools, to facilitate common processing steps for researchers who wish to use the NCPT data. The package is publicly available at https://github.com/pauljaffe/lumos-ncpt-tools/tree/v1.1.0. See the README file and “demo.ipynb” for a quick-start guide and example use cases. Among other features, the package has functions that enable inspecting the data (e.g. displaying summary statistics), filtering outlier scores, and filtering for participants who completed the entire test battery. For those that are more familiar with other programming languages, we note that packages are available that allow one to call Python commands from within other languages (e.g. the reticulate package for R and PyCall for Julia). Users should refer to the documentation for those packages for usage notes.

## Data Availability

All of the code used to generate the figures and perform the analyses are included with the public lumos-ncpt-tools repository described above: https://github.com/pauljaffe/lumos-ncpt-tools/tree/v1.1.0. See the README file for instructions on how to reproduce the figures and analyses. The software underlying the cognitive tasks themselves is proprietary and consequently cannot be shared at this time.
